# Supplementary material for: The β-TrCP-FBXW2-SKP2 axis regulates lung cancer cell growth with FBXW2 acting as a tumour suppressor
Source: Nat Commun. 2017 Jan 16;8:14002. doi: 10.1038/ncomms14002 (PMC5241824; doi:10.1038/ncomms14002)
Supplement: Supplementary Information — Supplementary Figures 1–8 and Supplementary Table 1 [file ncomms14002-s1.pdf]

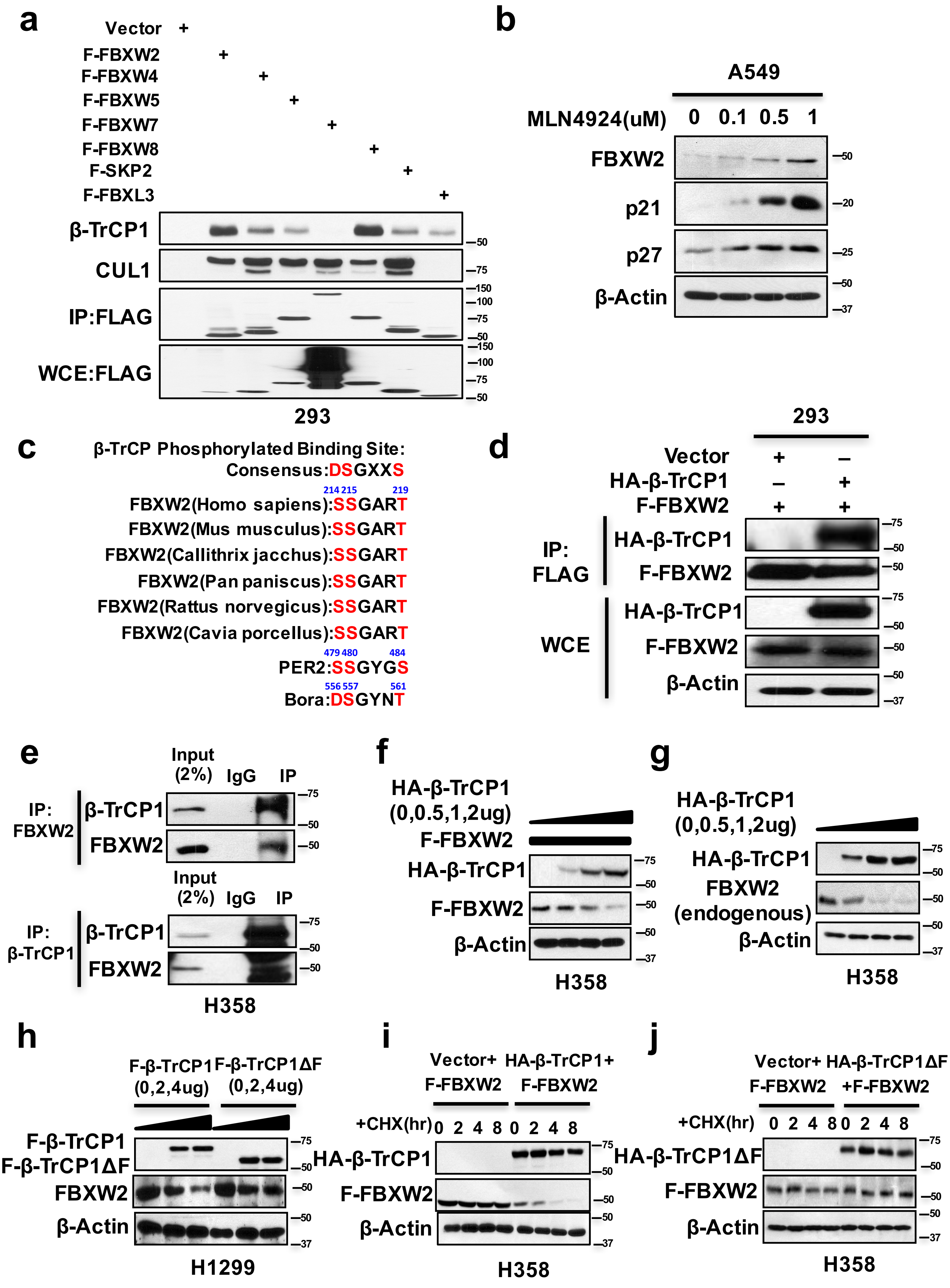
a
Vector
F-FBXW2
F-FBXW4
F-FBXW5
F-FBXW7
F-FBXW8
F-SKP2
F-FBXL3
β-TrCP1
CUL1
IP:FLAG
WCE:FLAG
293
b
A549
MLN4924(uM) 0 0.1 0.5 1
FBXW2
p21
p27
β-Actin
c
β-TrCP Phosphorylated Binding Site:
Consensus:DSGXXS
214 215 219
FBXW2(Homo sapiens):SSGART
FBXW2(Mus musculus):SSGART
FBXW2(Callithrix jacchus):SSGART
FBXW2(Pan paniscus):SSGART
FBXW2(Rattus norvegicus):SSGART
FBXW2(Cavia porcellus):SSGART
479 480 484
PER2:SSGYGS
556 557 561
Bora:DSGYNT
d
293
Vector + –
HA-β-TrCP1 – +
F-FBXW2 + +
IP: FLAG
HA-β-TrCP1
F-FBXW2
WCE
HA-β-TrCP1
F-FBXW2
β-Actin
e
Input (2%) IgG IP
IP: FBXW2
β-TrCP1
FBXW2
IP: β-TrCP1
β-TrCP1
FBXW2
H358
f
HA-β-TrCP1 (0,0.5,1,2ug)
F-FBXW2
HA-β-TrCP1
F-FBXW2
β-Actin
H358
g
HA-β-TrCP1 (0,0.5,1,2ug)
HA-β-TrCP1
FBXW2 (endogenous)
β-Actin
H358
h
F-β-TrCP1 (0,2,4ug)
F-β-TrCP1ΔF (0,2,4ug)
F-β-TrCP1
F-β-TrCP1ΔF
FBXW2
β-Actin
H1299
i
Vector+ F-FBXW2
HA-β-TrCP1+ F-FBXW2
+CHX(hr) 0 2 4 8 0 2 4 8
HA-β-TrCP1
F-FBXW2
β-Actin
H358
j
Vector+ F-FBXW2
HA-β-TrCP1ΔF +F-FBXW2
+CHX(hr) 0 2 4 8 0 2 4 8
HA-β-TrCP1ΔF
F-FBXW2
β-Actin
H358

Supplementary Figure 1. β-TrCP1 binds to FBXW2, regulates FBXW2 protein level and shortens FBXW2 Protein half-life.

(**a**) Binding of β-TrCP1 to seven F-box proteins: 293 cells were transfected with indicated plasmids, followed by FLAG-bead IP and IB with indicated Abs.

(**b**) FBXW2 accumulation by MLN4924 treatment: A549 cells were treated with MLN4924 in various concentrations, followed by IB with indicated Abs.

(c) Evolutionary conservation of β-TrCP binding motif on FBXW2.

(**d**) β-TrCP1 binds to exogenously expressed FBXW2: 293 cells were transfected with indicated plasmids, followed by FLAG-bead IP and IB with indicated Abs. WCE: whole cell extract.

(e) β-TrCP1 binds to endogenous FBXW2: Cell lysates from H358 cells were pulled down with anti-FBXW2 or anti-β-TrCP1 Abs, followed by IB with indicated Abs.

(f&g) β-TrCP1 overexpression decreases the levels of exogenous and endogenous FBXW2 proteins. H358 cells were co-transfected with FLAG-FBXW2 and increasing amounts of HA-β-TrCP1, or transfected with increasing amounts of HA-β-TrCP1 alone, followed by IB with indicated Abs.

(h) β-TrCP1ΔF fails to decrease FBXW2 protein level. H358 cells were transfected with increasing amounts of FLAG-β-TrCP or FLAG-β-TrCPΔF, and harvested for IB.

(i&j) β-TrCP1, but not β-TrCP1ΔF shorten FBXW2 protein half-life. H358 cells were transfected with FLAG-FBXW2, or in combination with HA-β-TrCP1 (i) or HA-β-TrCP1ΔF (j). Cells were switched 48 hrs post transfection to fresh medium containing CHX for indicated time periods and harvested for IB.

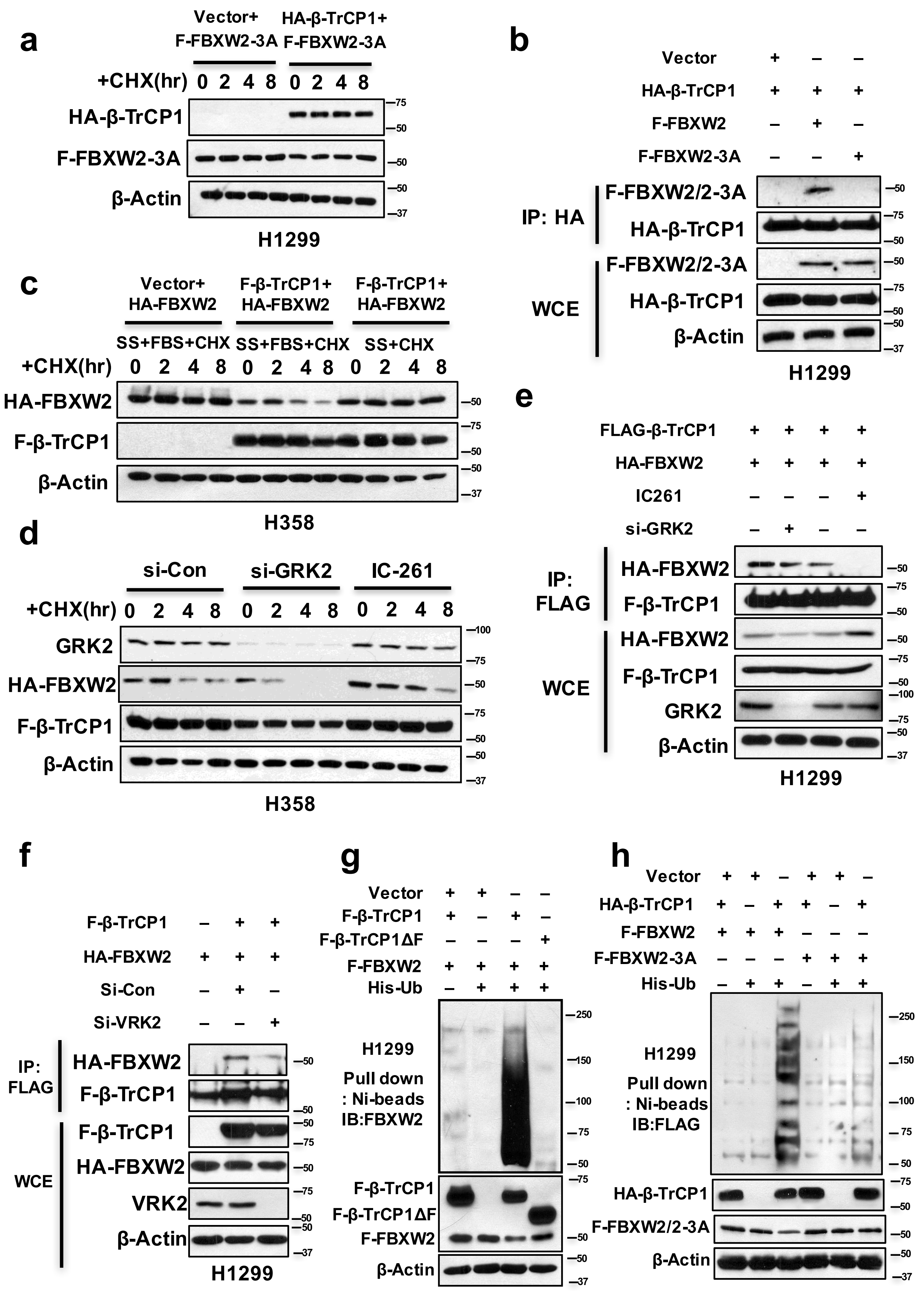
a
Vector+ F-FBXW2-3A
HA-β-TrCP1+ F-FBXW2-3A
+CHX(hr) 0 2 4 8 0 2 4 8
HA-β-TrCP1
F-FBXW2-3A
β-Actin
H1299
b
Vector + – –
HA-β-TrCP1 + + +
F-FBXW2 – + –
F-FBXW2-3A – – +
IP: HA
F-FBXW2/2-3A
HA-β-TrCP1
WCE
F-FBXW2/2-3A
HA-β-TrCP1
β-Actin
H1299
c
Vector+ HA-FBXW2
F-β-TrCP1+ HA-FBXW2
F-β-TrCP1+ HA-FBXW2
SS+FBS+CHX SS+FBS+CHX SS+CHX
+CHX(hr) 0 2 4 8 0 2 4 8 0 2 4 8
HA-FBXW2
F-β-TrCP1
β-Actin
H358
d
si-Con si-GRK2 IC-261
+CHX(hr) 0 2 4 8 0 2 4 8 0 2 4 8
GRK2
HA-FBXW2
F-β-TrCP1
β-Actin
H358
e
FLAG-β-TrCP1 + + + +
HA-FBXW2 + + + +
IC261 – – – +
si-GRK2 – + – –
IP: FLAG
HA-FBXW2
F-β-TrCP1
WCE
HA-FBXW2
F-β-TrCP1
GRK2
β-Actin
H1299
f
F-β-TrCP1 – + +
HA-FBXW2 + + +
Si-Con – + –
Si-VRK2 – – +
IP: FLAG
HA-FBXW2
F-β-TrCP1
WCE
F-β-TrCP1
HA-FBXW2
VRK2
β-Actin
H1299
g
Vector + + – –
F-β-TrCP1 + – + –
F-β-TrCP1ΔF – – – +
F-FBXW2 + + + +
His-Ub – + + +
H1299
Pull down : Ni-beads IB:FBXW2
F-β-TrCP1
F-β-TrCP1ΔF
F-FBXW2
β-Actin
h
Vector + + – + + –
HA-β-TrCP1 + – + + – +
F-FBXW2 + + + – – –
F-FBXW2-3A – – – + + +
His-Ub – + + – + +
H1299
Pull down : Ni-beads IB:FLAG
HA-β-TrCP1
F-FBXW2/2-3A
β-Actin

Supplementary Figure 2. β-TrCP1 binds to FBXW2 via a "SSGART" motif and promotes FBXW2 ubiquitylation.

(a&b) βTrCP1 failed to bind to or shorten protein half-life of FBXW2-3A mutant. H1299 cells were transfected with indicated plasmids. Cells were then treated with CHX and harvested at indicated time points for IB (a), or pulled down with anti-HA Ab and IB with indicated Abs (b). WCE: whole cell extracts.

(c) β-TrCP1 shortens the protein half-life of exogenous FBXW2, triggered by serum addition to serum-starved cells: HA-FBXW2 in combination with, the vector control or FLAG-β-TrCP1, was co-transfected into H358 cells. Cells were switched 48 hrs post transfection to fresh medium containing10% FBS and CHX for indicated time periods and harvested for IB. SS: serum starvation, FBS: fetal bovine serum.

(d) CK1 kinase mediates FBXW2 phosphorylation at the β-TrCP1 binding motif: H358 cells were transfected of siRNA targeting GRK2, or treated by CK1 inhibitor IC-261 (10 μM), followed by transfected with FLAG-β-TrCP1 and HA-FBXW2 for 48 hrs. Cells were harvested at indicated time points after CHX treatment for IB with indicated Abs.

(e&f) CK1 inhibitor or VRK2 knockdown disrupts βTrCP1-FBXW2 binding. H1299 cells was transfected with siRNA targeting GRK2 or treated by CK1 inhibitor IC-261 (10 μM) (e), or transfected with siRNA targeting VRK2 (f), followed by co-transfected with FLAG-β-TrCP1 and HA-FBXW2 for 48hrs. Cells were harvested at indicated time points after CHX treatment for IB with indicated Abs.

(g&h) β-TrCP1, but not β-TrCP1ΔF promotes FBXW2 ubiquitylation: H1299 cells were transfected with indicated plasmids, lysed under denatured condition at 6M guanidinium solution, followed by Ni-beads pull-down. Washed beads were boiled for IB to detect polyubiquitylation of FBXW2.

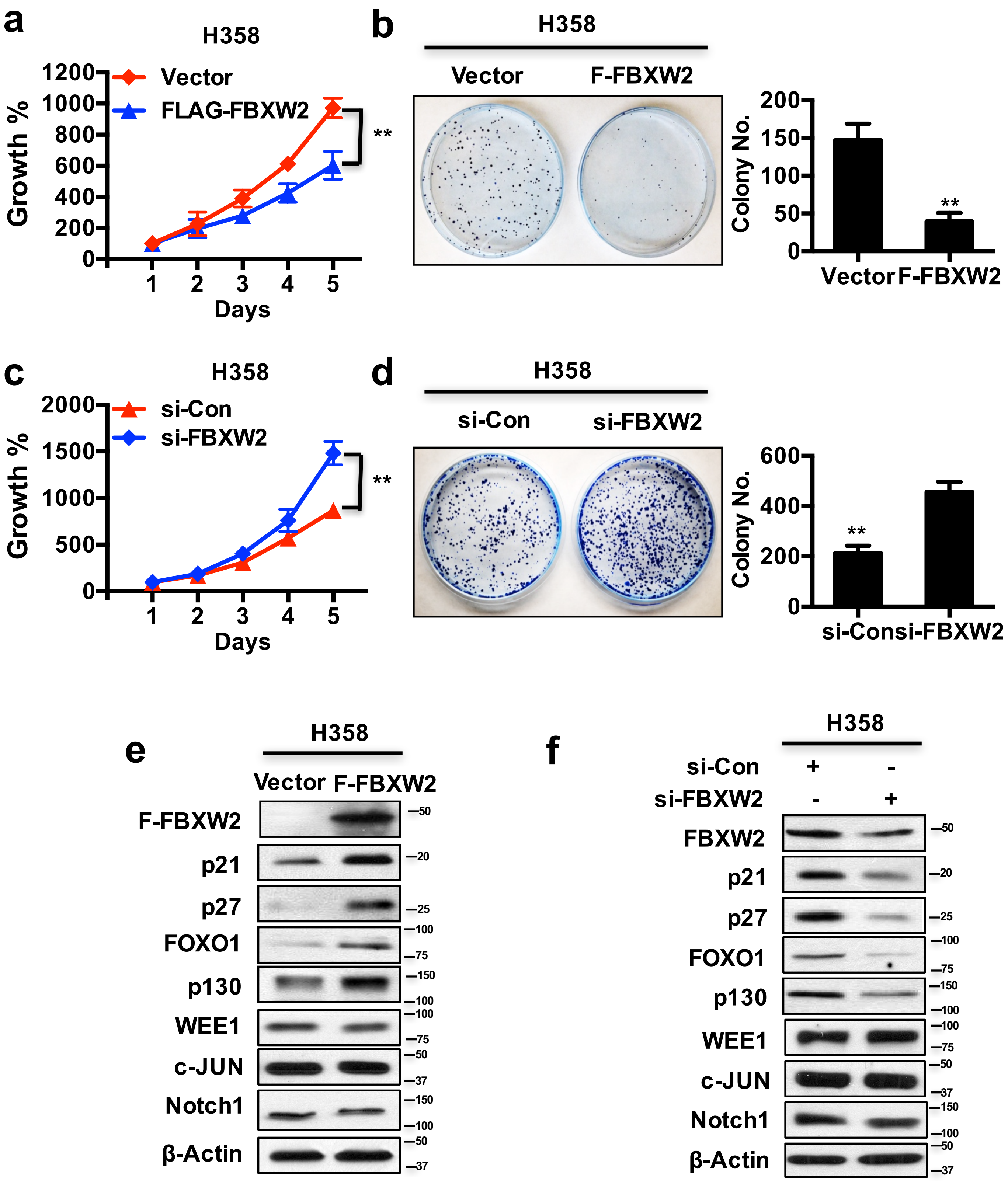
a
H358
Growth %
Vector
FLAG-FBXW2
**
Days
b
H358
Vector
F-FBXW2
Colony No.
**
Vector
F-FBXW2
c
H358
Growth %
si-Con
si-FBXW2
**
Days
d
H358
si-Con
si-FBXW2
Colony No.
**
si-Con
si-FBXW2
e
H358
Vector
F-FBXW2
F-FBXW2
p21
p27
FOXO1
p130
WEE1
c-JUN
Notch1
β-Actin
f
H358
si-Con
si-FBXW2
FBXW2
p21
p27
FOXO1
p130
WEE1
c-JUN
Notch1
β-Actin

Supplementary Figure 3. FBXW2 suppresses cell growth and survival in H358 cells

(a-d) FBXW2 overexpression inhibits, but siRNA silencing promotes growth and survival of lung cancer cells. H358 cells were transfected with FLAG-FBXW2 (a&b) or silenced by siRNA targeting FBXW2 (c&d), followed by ATP-lite proliferation assay (n=3) (a&c), and clonogenic survival (n=3) (b&d). Shown is X ± SEM. Student t test was performed, * $p < 0.05$; ** $p < 0.01$.

(e&f) FBXW2 overexpression increases, whereas siRNA silencing decreases the levels of tumor suppressor proteins: H358 cells were transfected with FLAG-FBXW2 (e), or silenced with siRNA targeting FBXW2 (f), followed by IB with indicated Abs.

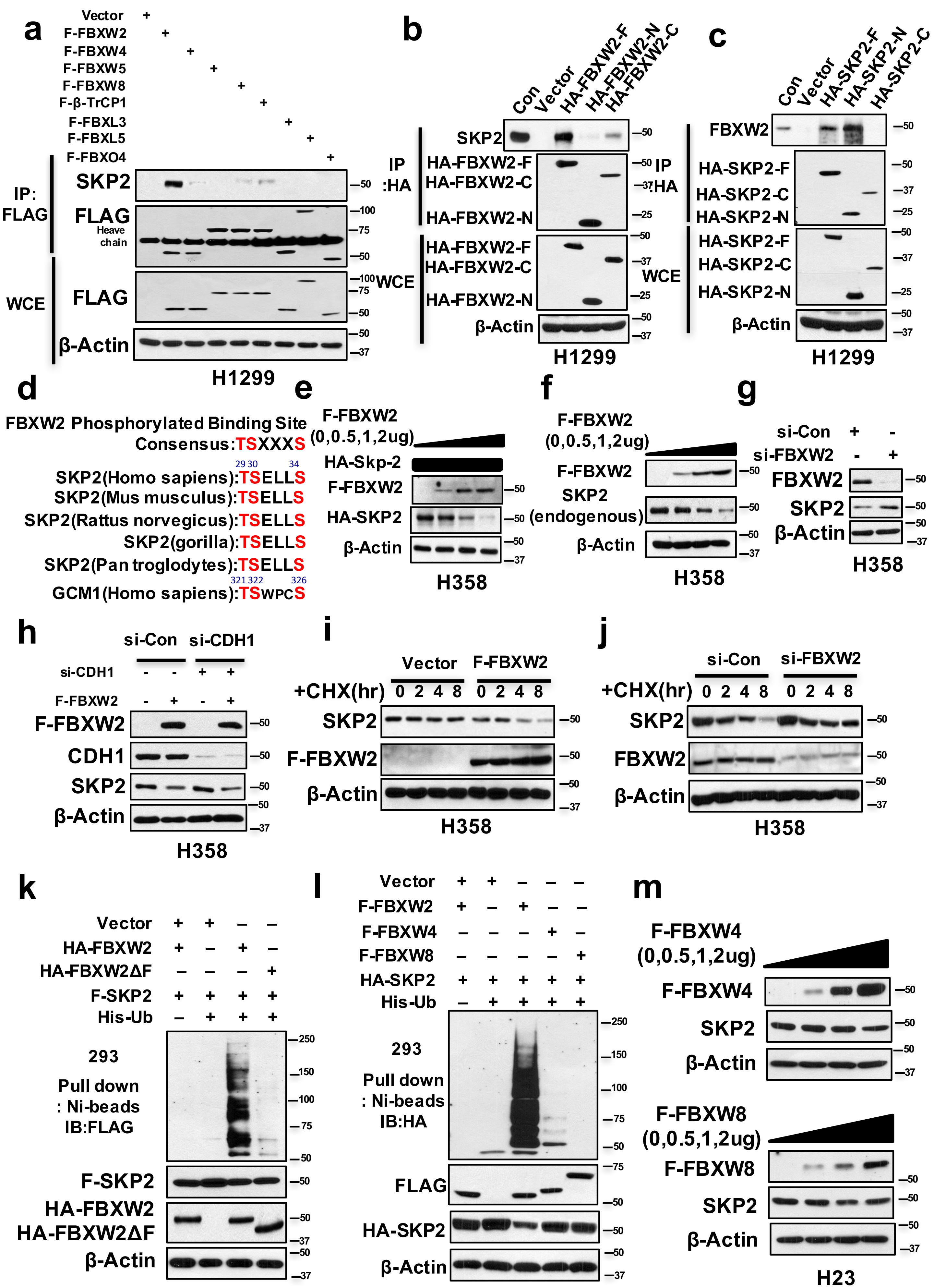

a
Vector
F-FBXW2
F-FBXW4
F-FBXW5
F-FBXW8
F-β-TrCP1
F-FBXL3
F-FBXL5
F-FBXO4
IP: FLAG
SKP2
FLAG
Heave chain
WCE
FLAG
β-Actin
H1299
b
Con
Vector
HA-FBXW2-F
HA-FBXW2-N
HA-FBXW2-C
IP :HA
SKP2
HA-FBXW2-F
HA-FBXW2-C
HA-FBXW2-N
WCE
β-Actin
H1299
c
HA-SKP2-F
HA-SKP2-N
HA-SKP2-C
FBXW2
HA-SKP2-F
HA-SKP2-C
HA-SKP2-N
β-Actin
H1299
d
FBXW2 Phosphorylated Binding Site
Consensus:TSXXXS
SKP2(Homo sapiens):TSELLS
SKP2(Mus musculus):TSELLS
SKP2(Rattus norvegicus):TSELLS
SKP2(gorilla):TSELLS
SKP2(Pan troglodytes):TSELLS
GCM1(Homo sapiens):TSWPCS
e
F-FBXW2 (0,0.5,1,2ug)
HA-Skp-2
F-FBXW2
HA-SKP2
β-Actin
H358
f
F-FBXW2 (0,0.5,1,2ug)
F-FBXW2
SKP2 (endogenous)
β-Actin
H358
g
si-Con
si-FBXW2
FBXW2
SKP2
β-Actin
H358
h
si-Con
si-CDH1
si-CDH1
F-FBXW2
F-FBXW2
CDH1
SKP2
β-Actin
H358
i
Vector
F-FBXW2
+CHX(hr)
SKP2
F-FBXW2
β-Actin
H358
j
si-Con
si-FBXW2
+CHX(hr)
SKP2
FBXW2
β-Actin
H358
k
Vector
HA-FBXW2
HA-FBXW2ΔF
F-SKP2
His-Ub
293
Pull down : Ni-beads IB:FLAG
F-SKP2
HA-FBXW2
HA-FBXW2ΔF
β-Actin
l
Vector
F-FBXW2
F-FBXW4
F-FBXW8
HA-SKP2
His-Ub
293
Pull down : Ni-beads IB:HA
FLAG
HA-SKP2
β-Actin
m
F-FBXW4 (0,0.5,1,2ug)
F-FBXW4
SKP2
β-Actin
F-FBXW8 (0,0.5,1,2ug)
F-FBXW8
SKP2
β-Actin
H23

Supplementary Figure 4. FBXW2 binds to SKP2 and promotes its ubiquitylation and degradation

(a) SKP2 mainly binds to FBXW2. H1299 cells were transfected with indicated plasmid, followed by IP with FLAG-Ab and IB with SKP2 Ab.

(b-c) Mapping the binding domains between FBXW2 and SKP2. H1299 cells were transfected with indicated plasmids expressing wild type or truncated proteins: FBXW2-F (full length, codons 1-454), FBXW2-N (N-terminus, codons 1-138); FBXW2-C (C-terminus, codons 139-454); SKP2-F (full length, codons 1-358), SKP2-N (N-terminus, codons 1-150), and SKP2-C (C-terminus, codon 151-358), followed by IP with HA-Ab and IB with antibodies against SKP2 (b) or FBXW2 (c). Expression of each plasmid was shown in the bottom panel.

(d) Evolutionary conservation of FBXW2 binding motif on SKP2.

(e&f) FBXW2 overexpression decreases the levels of the exogenous and endogenous SKP2 proteins: H358 cells were co-transfected with HA-SKP2 and increasing amount of FLAG-FBXW2 (e), or transfected with increasing amount of HA-FBXW2 alone (f), followed by IB with indicated Abs 48 hrs post transfection.

(g) FBXW2 depletion increases the levels of SKP2 protein. H358 cells were transfected with FBXW2 siRNA and scramble siRNA control, followed by IB.

(h) FBXW2-induced SKP2 degradation is independent of CDH1. H358 cells were transfected with FLAG-FBXW2 and/or siRNA against CDH1, followed by IB.

(i&j) FBXW2 overexpression shortens but its siRNA silencing extends SKP2 half-life. H358 cells were transfected with FLAG-FBXW2 (i), or FBXW2 siRNA (j), followed by CHX treatment and subsequent cell collection at indicated time for IB.

(k&l) The 293 cells were transfected with indicated plasmids, followed by Ni-bead pull-down. Washed beads were boiled and subjected to IB with Abs against FLAG (k) or HA (l).

(m) FBXW4 and FBXW8 overexpression cannot shorten SKP2 half-life. H23 cells were transfected with increasing amounts of FLAG-FBXW4 and FLAG-FBXW8, followed by IB 48hrs post-transfection.

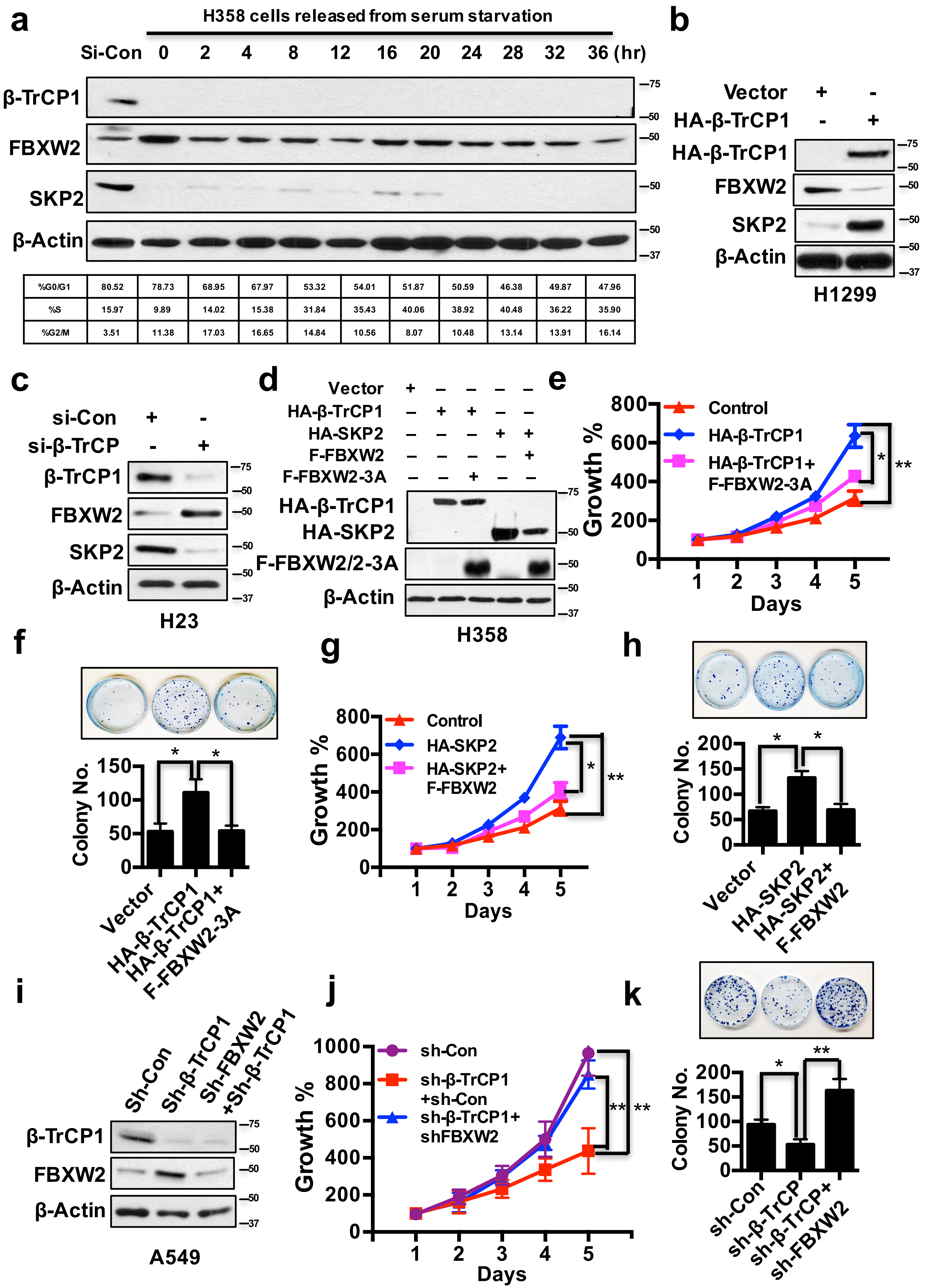

	Si-Con	0	2	4	8	12	16	20	24	28	32
%G0/G1	80.52	78.73	68.95	67.97	53.32	54.01	51.87	50.59	46.38	49.87	47.96
%S	15.97	9.89	14.02	15.38	31.84	35.43	40.06	38.92	40.48	36.22	35.90
%G2/M	3.51	11.38	17.03	16.65	14.84	10.56	8.07	10.48	13.14	13.91	16.14

Supplementary Figure 5. FBXW2 mediates the biological effects of β-TrCP1 as a downstream effector and of SKP2 as an upstream modulator

(a) Fluctuation of the levels of F-box proteins during cell cycle progression upon β-TrCP silencing: H358 cells were transfected with siRNA targeting β-TrCP, followed by serum starved for 48 hrs and then serum addition. Cells were harvested at indicated time points and subjected to FACS and IB analyses using indicated Abs.

(**b&c**) Ectopically expressed β-TrCP1 reduces the endogenous levels of FBXW2 and increases the endogenous levels of SKP2, and β-TrCP depletion increases the endogenous levels of FBXW2 and reduces the endogenous levels of SKP2. Cells were transfected with HA-β-TrCP1 (b), or with siRNA targeting both β-TrCP1 and β-TrCP2 (c), along with scramble siRNA control, followed by IB.

(d-h) FBXW2-3A mutant rescues growth-promoting phenotype induced by β-TrCP1 overexpression (d-f), and wt FBXW2 rescues growth-promoting phenotype induced by SKP2 overexpression (d and g&h): H358 cells were co-transfected with the indicated plasmids, followed by IB (d), ATP-lite proliferation assay (n = 3) (e&g), and clonogenic survival assay (n = 3) (f&h). Shown is X ± SEM. Student *t* test was performed, * $p < 0.05$; ** $p < 0.01$.

(i-k) β-TrCP1 depletion suppresses cell growth, which is abrogated by simultaneous FBXW2 depletion: A549 cells were transfected with shRNAs targeting β-TrCP1 alone or in combination with shRNA targeting FBXW2, along with scramble control, and then harvested for IB (i), ATP-lite proliferation assay (n = 3) (j), and clonogenic survival assay (n = 3) (k). Shown is X ± SEM. Student t test was performed, * $p < 0.05$; ** $p < 0.01$.

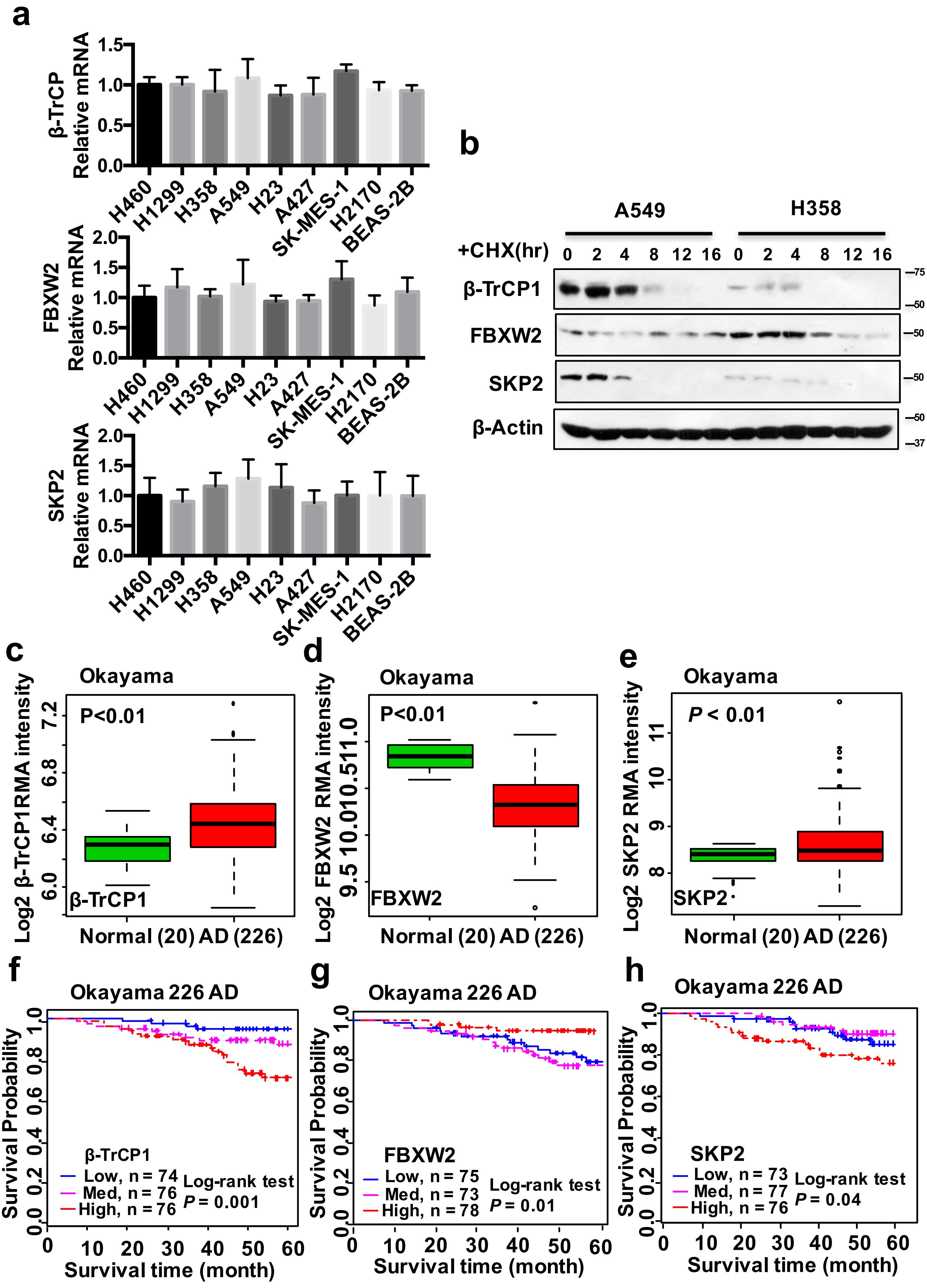
a
β-TrCP Relative mRNA
FBXW2 Relative mRNA
SKP2 Relative mRNA
H460 H1299 H358 A549 H23 A427 SK-MES-1 H2170 BEAS-2B
b
A549
H358
+CHX(hr) 0 2 4 8 12 16 0 2 4 8 12 16
β-TrCP1
FBXW2
SKP2
β-Actin
c
Okayama
P<0.01
Log2 β-TrCP1RMA intensity
β-TrCP1
Normal (20) AD (226)
d
Okayama
P<0.01
Log2 FBXW2 RMA intensity
FBXW2
Normal (20) AD (226)
e
Okayama
P < 0.01
Log2 SKP2 RMA intensity
SKP2
Normal (20) AD (226)
f
Okayama 226 AD
Survival Probability
β-TrCP1
Low, n = 74
Med, n = 76
High, n = 76
Log-rank test
P = 0.001
Survival time (month)
g
Okayama 226 AD
Survival Probability
FBXW2
Low, n = 75
Med, n = 73
High, n = 78
Log-rank test
P = 0.01
Survival time (month)
h
Okayama 226 AD
Survival Probability
SKP2
Low, n = 73
Med, n = 77
High, n = 76
Log-rank test
P = 0.04
Survival time (month)

Supplementary Figure 6. The mRNA expression of FBXW2, β-TrCP1 and SKP2 in lung cancer tissues and their association with patient survival.

(a) Expression of FBXW2, β-TrCP1 and SKP2 mRNAs. Total RNA was isolated from indicated lung cancer cell lines and subjected to qRT-PCR analysis.

(b) Protein half-life of FBXW2, β-TrCP1 and SKP2. Cell lysates were prepared from two indicated lung cancer cell lines after CHX treatment for an indicated period of time and subjected to IB for indicated Abs.

(c-h) Continuous gene expression values were classified in to low, medium and high groups with equal number of patients, and 5-year survival time was used for Kaplan-Meier survival analysis. Boxplot and Kaplan-Meier survival analysis indicated that FBXW2 mRNA expression was decreased in lung cancer as compared to normal lung tissues (t test, $p < 0.01$) (d), and this lower expression was related to a worse overall patient survival (log-rank test, $p = 0.01$) (g); β-TrCP1 and SKP2 mRNA expression were increased in tumors as compared to normal (t test, $p < 0.01$) (c&e), and increased expression were related to a worse overall patient survival (log-rank test, $p = 0.001$ and 0.04, respectively) (f&h).

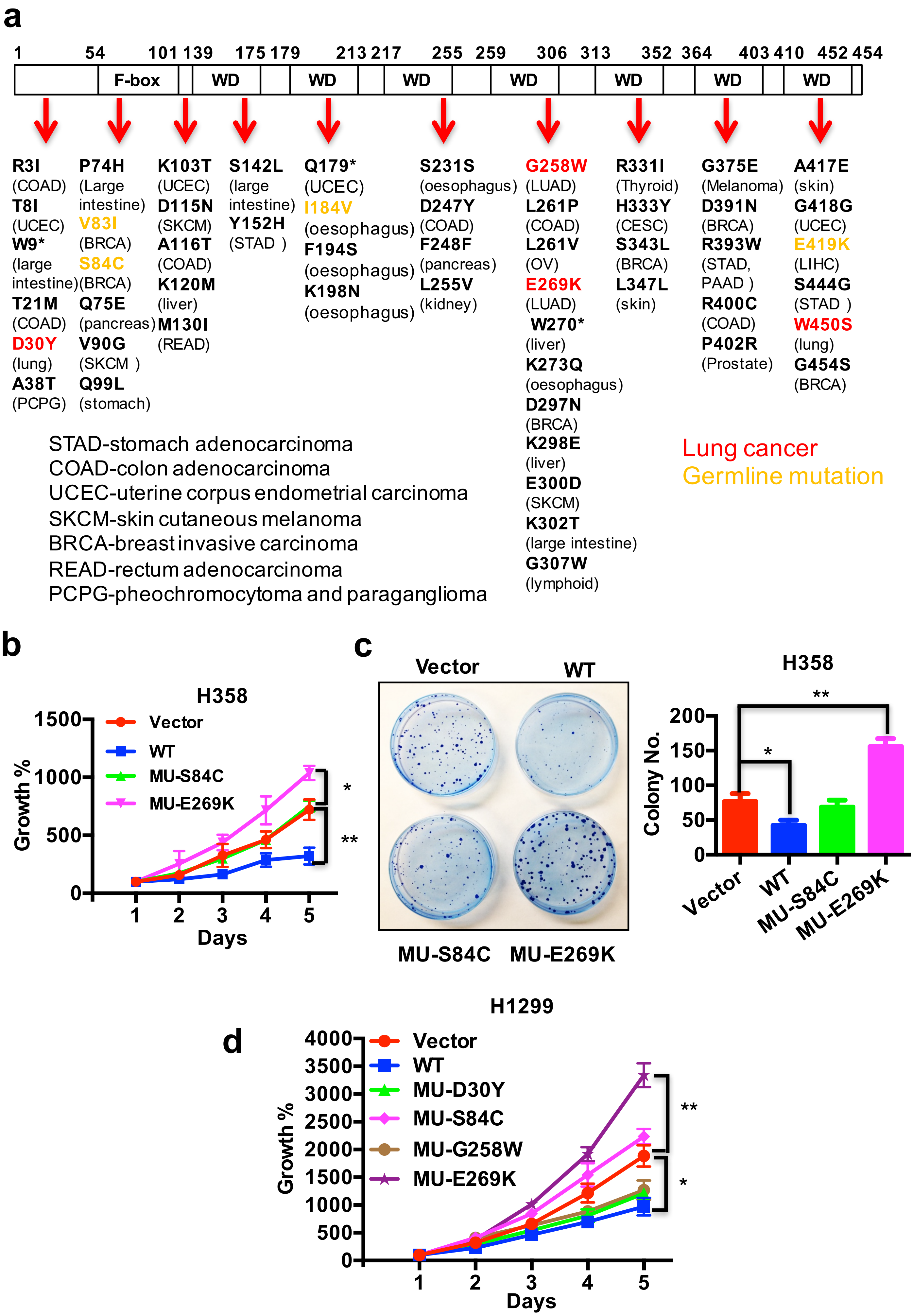
a
1 54 101 139 175 179 213 217 255 259 306 313 352 364 403 410 452 454
F-box
WD
WD
WD
WD
WD
WD
WD
R3I (COAD) T8I (UCEC) W9* (large intestine) T21M (COAD) D30Y (lung) A38T (PCPG)
P74H (Large intestine) V83I (BRCA) S84C (BRCA) Q75E (pancreas) V90G (SKCM) Q99L (stomach)
K103T (UCEC) D115N (SKCM) A116T (COAD) K120M (liver) M130I (READ)
S142L (large intestine) Y152H (STAD)
Q179* (UCEC) I184V (oesophagus) F194S (oesophagus) K198N (oesophagus)
S231S (oesophagus) D247Y (COAD) F248F (pancreas) L255V (kidney)
G258W (LUAD) L261P (COAD) L261V (OV) E269K (LUAD) W270* (liver) K273Q (oesophagus) D297N (BRCA) K298E (liver) E300D (SKCM) K302T (large intestine) G307W (lymphoid)
R331I (Thyroid) H333Y (CESC) S343L (BRCA) L347L (skin)
G375E (Melanoma) D391N (BRCA) R393W (STAD, PAAD) R400C (COAD) P402R (Prostate)
A417E (skin) G418G (UCEC) E419K (LIHC) S444G (STAD) W450S (lung) G454S (BRCA)
STAD-stomach adenocarcinoma
COAD-colon adenocarcinoma
UCEC-uterine corpus endometrial carcinoma
SKCM-skin cutaneous melanoma
BRCA-breast invasive carcinoma
READ-rectum adenocarcinoma
PCPG-pheochromocytoma and paraganglioma
Lung cancer
Germline mutation
b
H358
Growth %
1500
1000
500
0
Vector
WT
MU-S84C
MU-E269K
*
**
1 2 3 4 5
Days
c
Vector
WT
MU-S84C
MU-E269K
H358
Colony No.
200
150
100
50
0
**
*
Vector
WT
MU-S84C
MU-E269K
d
H1299
Growth %
4000
3500
3000
2500
2000
1500
1000
500
0
Vector
WT
MU-D30Y
MU-S84C
MU-G258W
MU-E269K
**
*
1 2 3 4 5
Days

Supplementary Figure 7. Distribution of FBXW2 mutations found in human cancers and their loss-of-function and gain-of-function activities in lung cancer cells.

(a) FBXW2 mutations distribution in various human cancers. The mutations with abbreviation of cancer tissue types were from TCGA database, whereas the remaining with tissue types spelled-out were from COSMIC database.

(b-c) Loss- or gain-of-function of FBXW2 mutants. H358 cells were transfected with indicated plasmids, followed by G418 selection. Resistant clones were pooled and followed by ATP-lite proliferation assay (n = 3) (b) and clonogenic survival assay (n = 3) (c). Shown is X ± SEM. Student t test was performed, * $p < 0.05$; ** $p < 0.01$.

(d) Effect of FBXW2 mutants on cell growth. H1299 cells were transfected with indicated plasmids, followed by G418 selection. Resistant clones were pooled and followed by ATP-lite proliferation assay (n = 3). Shown is X ± SEM. Student t test was performed, * $p < 0.05$; ** $p < 0.01$.

Supplementary Fig. 8

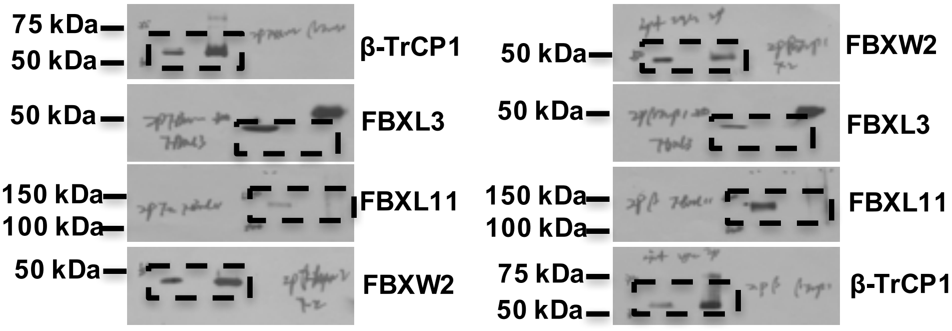

Figure 1a

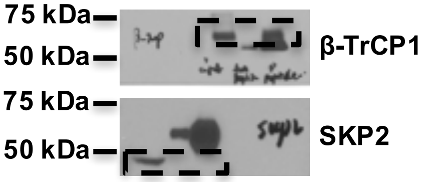

Figure 1b

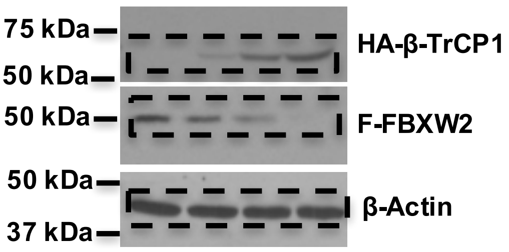

Figure 1c

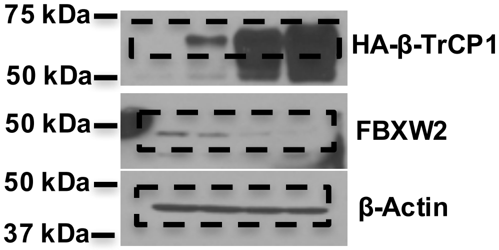

Figure 1d

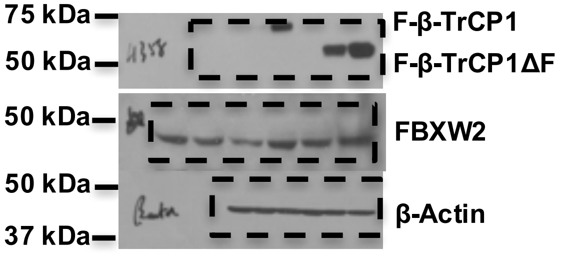

Figure 1e

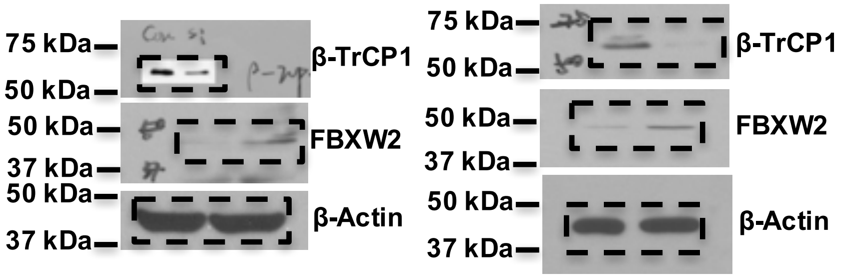

Figure 1g

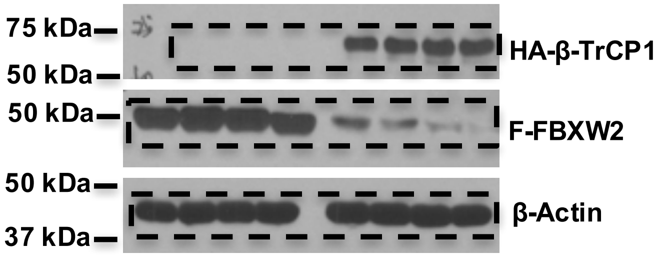

Figure 1h

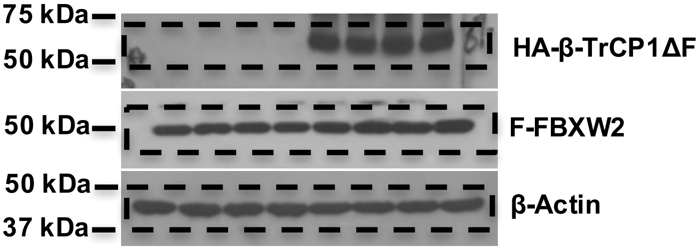

Figure 1i

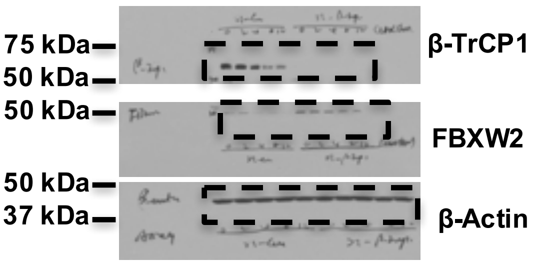

Figure 1j

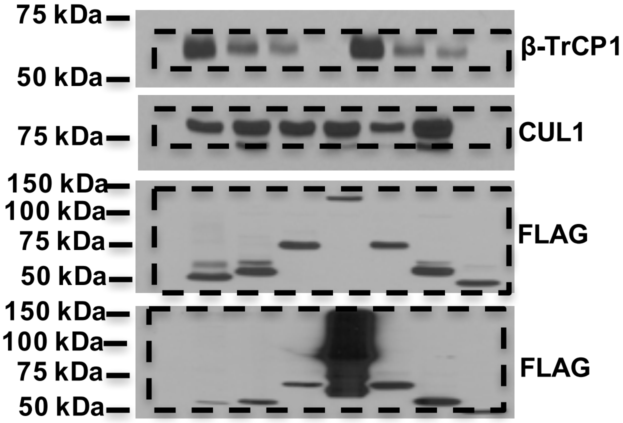

Figure s1a

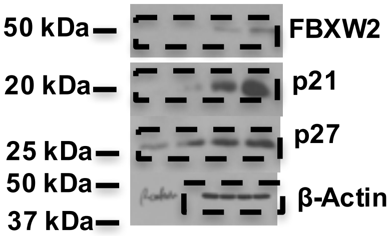

Figure s1b

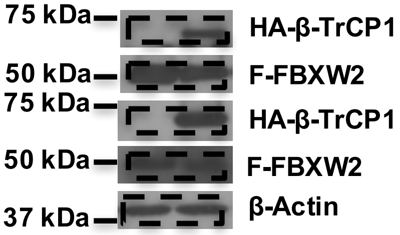

Figure s1d

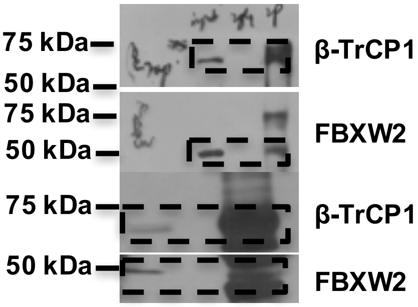

Figure s1e

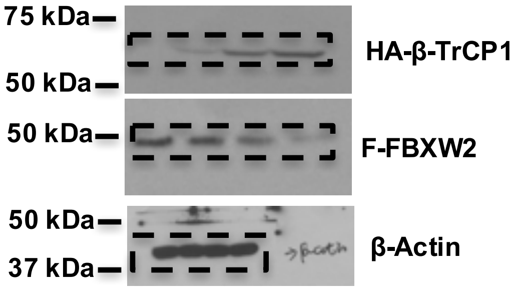

Figure s1f

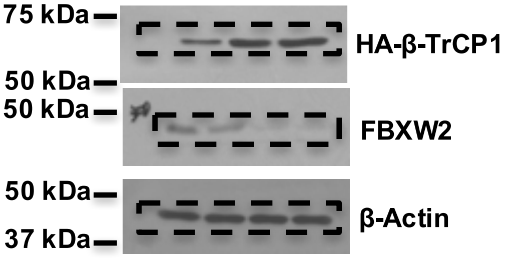

Figure s1g

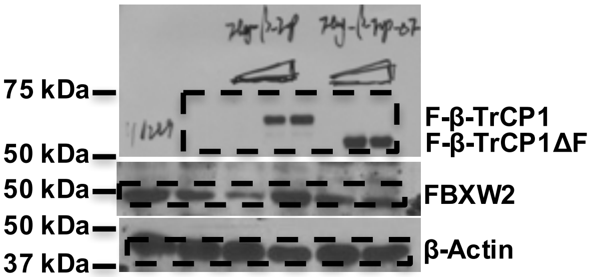

Figure s1h

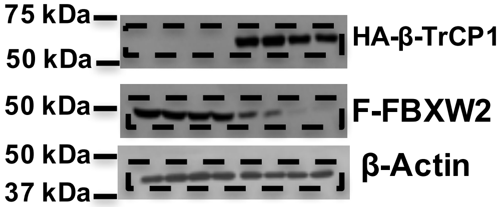

Figure s1i

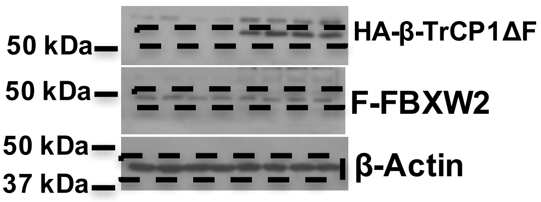

Figure s1j

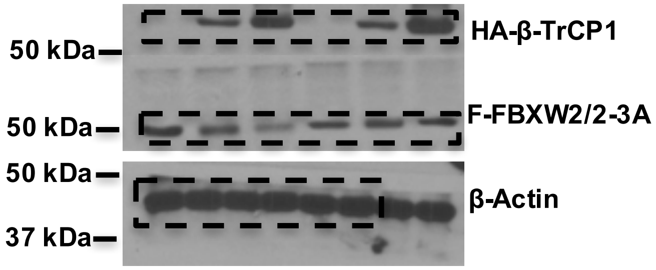

Figure 2a

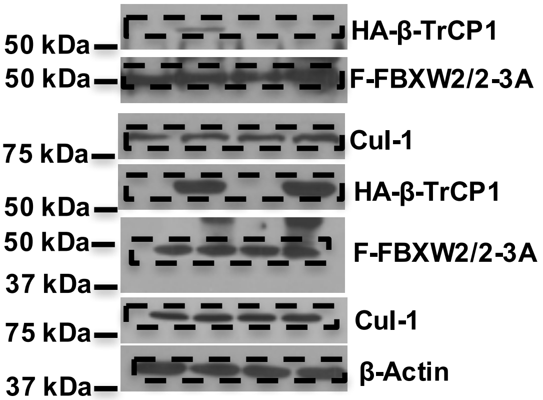

Figure 2b

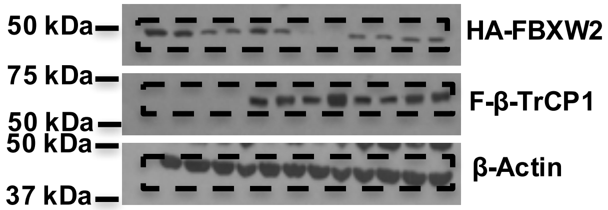

Figure 2c

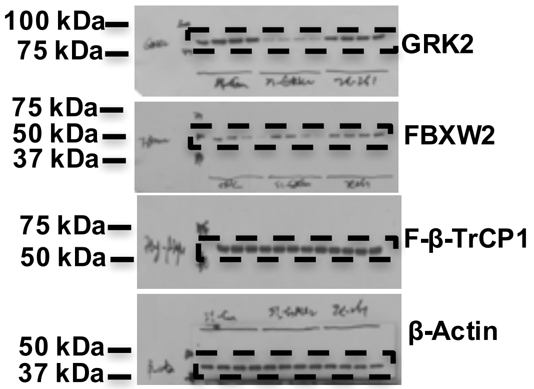

Figure 2d

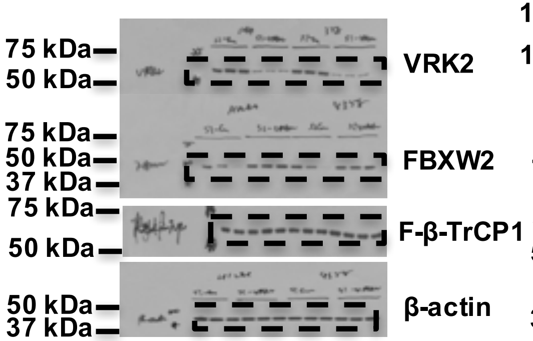

Figure 2e

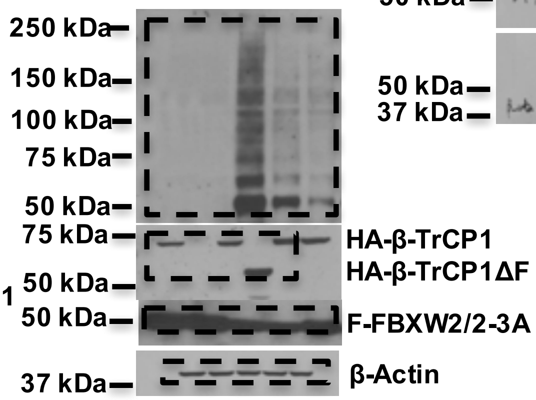

Figure 2f

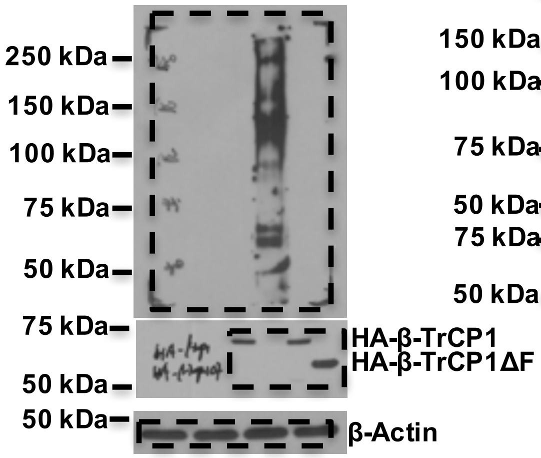

Figure 2g

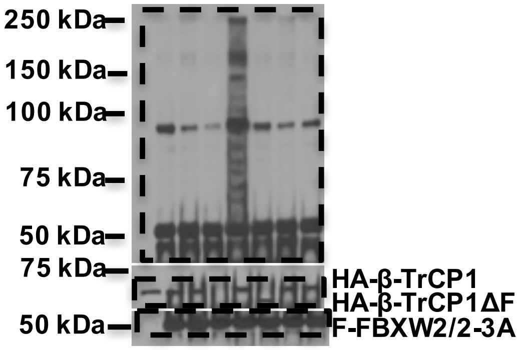

Figure 2h

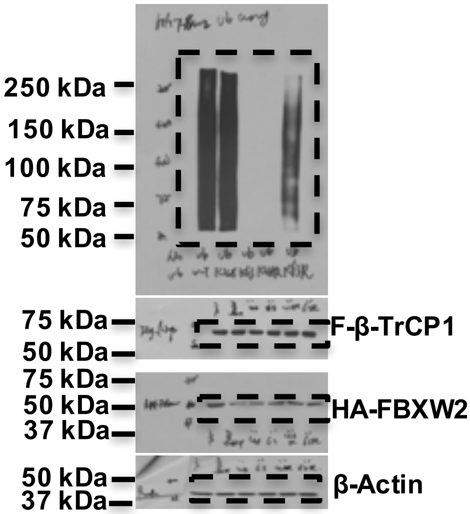

Figure 2i

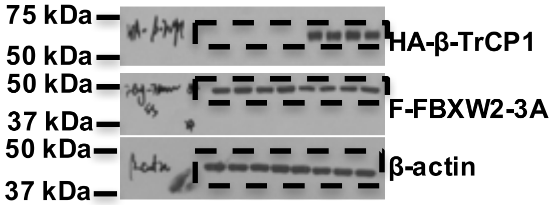

Figure s2a

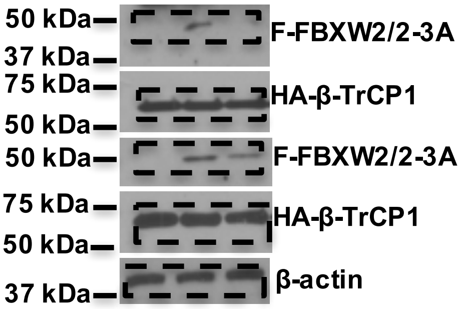

Figure s2b

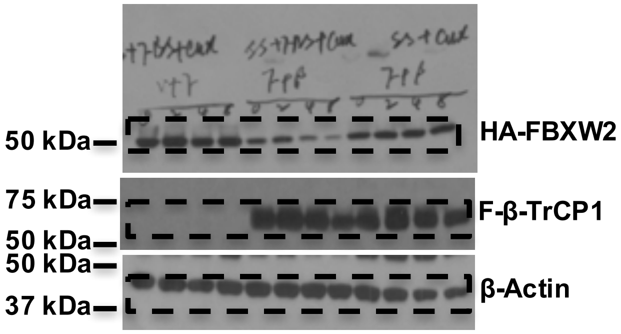

Figure s2c

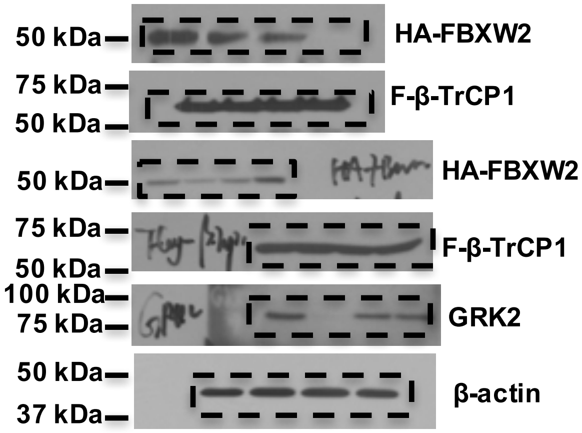

Figure s2e

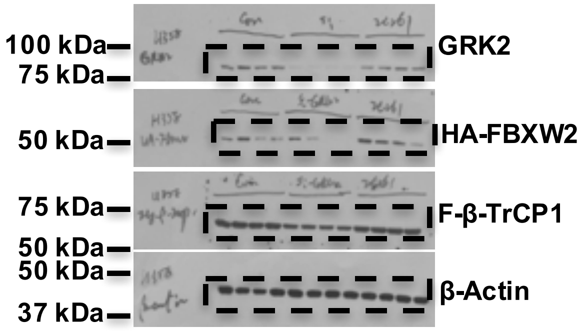

Figure s2d

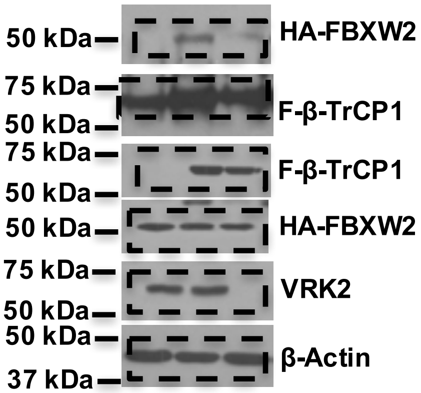

Figure s2f

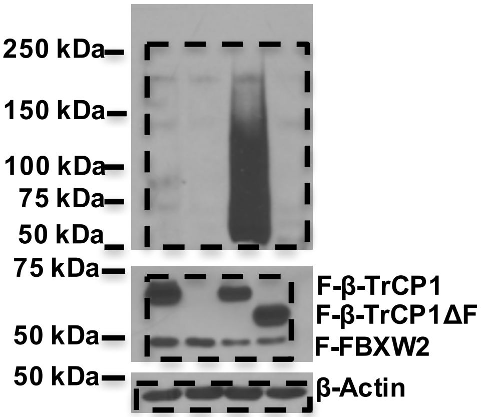

Figure s2g

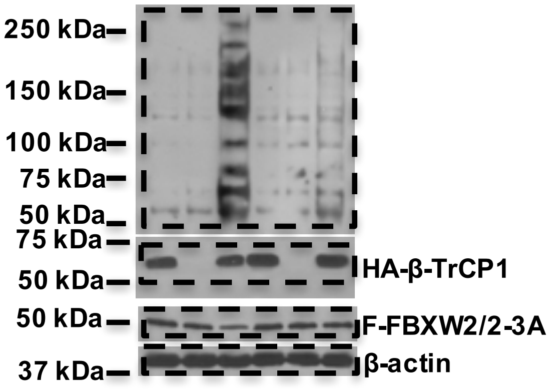

Figure s2h

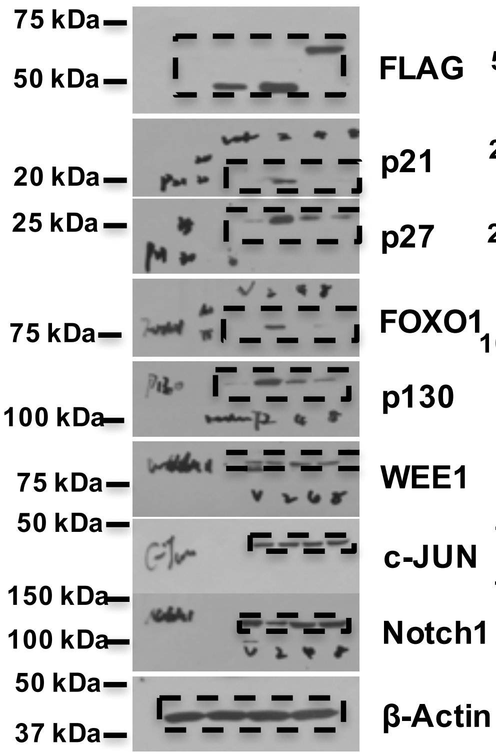

Figure 3g

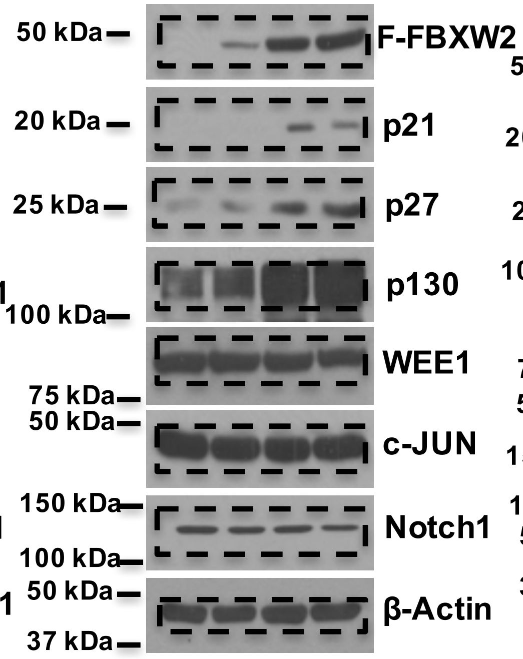

Figure 3h

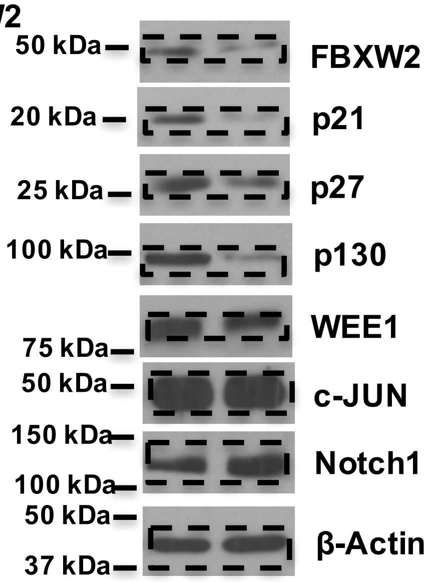

Figure 3i

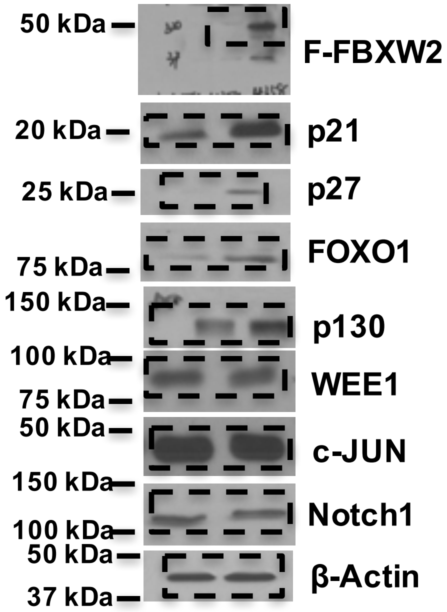

Figure s3e

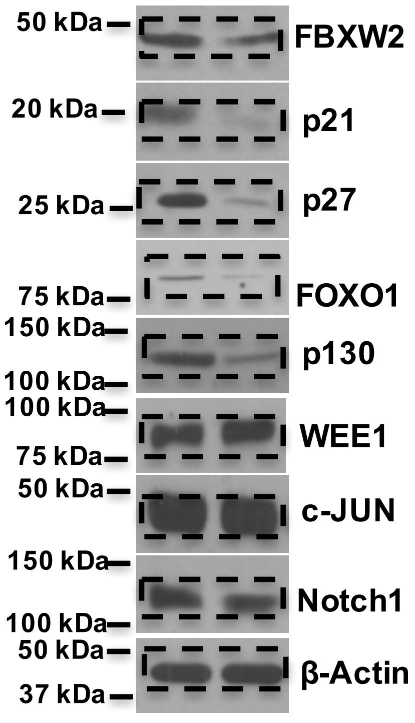

Figure s3f

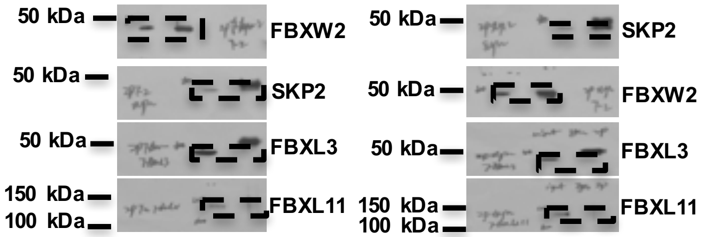

Figure 4a

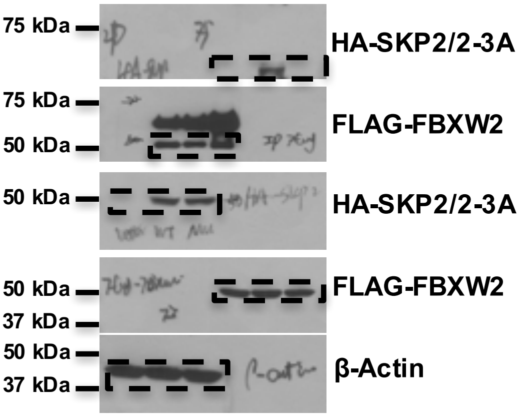

Figure 4b

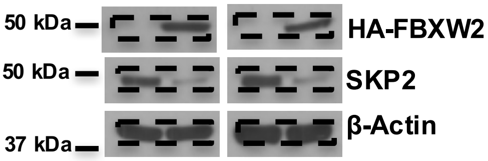

Figure 4c

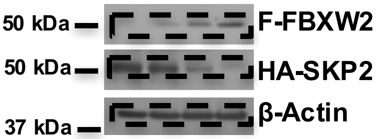

Figure 4e

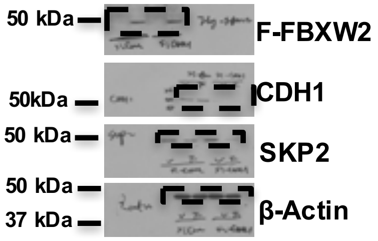

Figure 4h

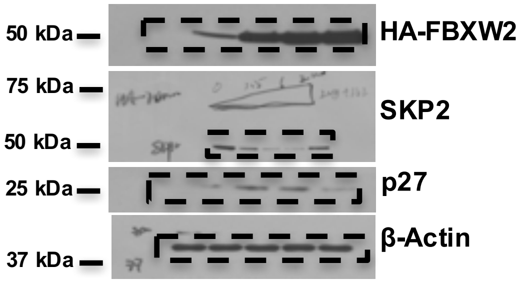

Figure 4f

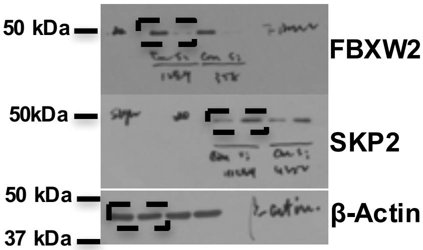

Figure 4g

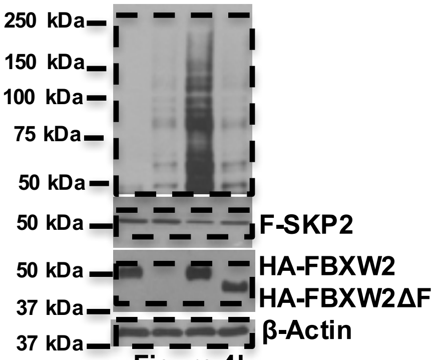

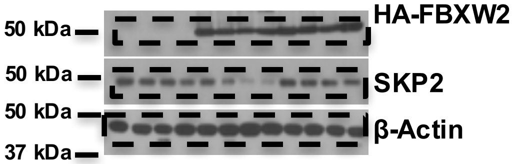

Figure 4i

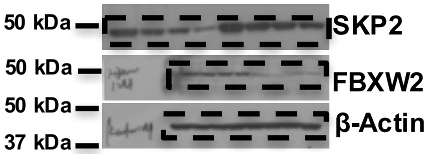

Figure 4j

Figure 4k

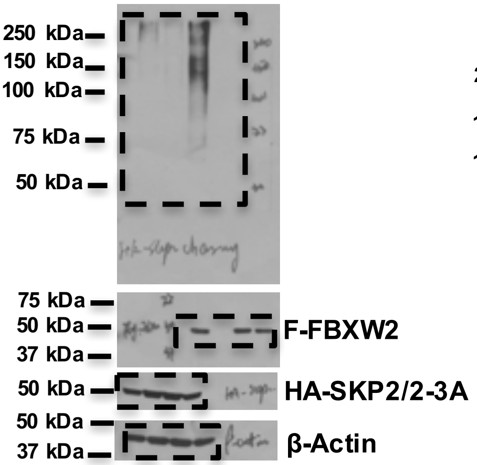

Figure 4l

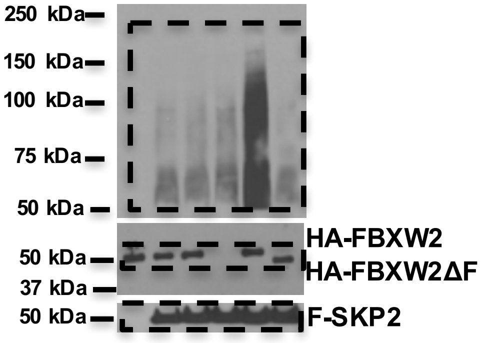

Figure 4m

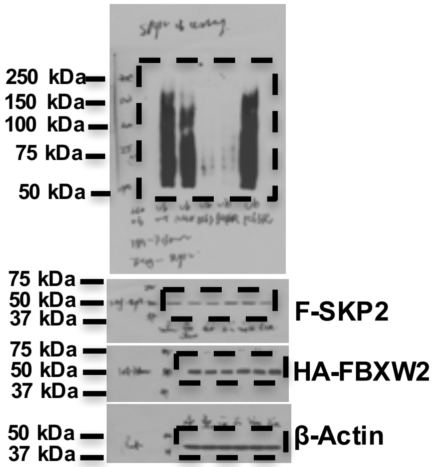

Figure 4n

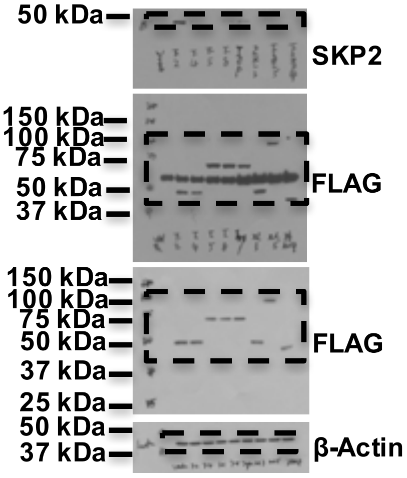

Figure s4a

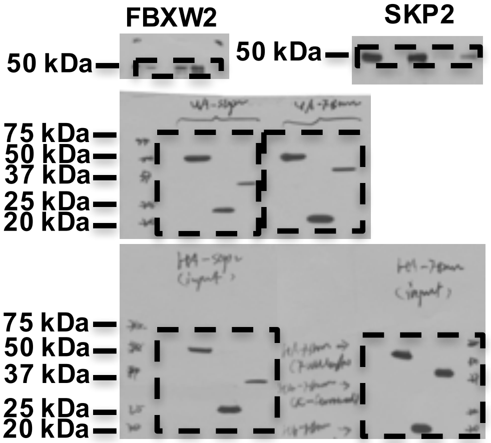

Figure s4b and c

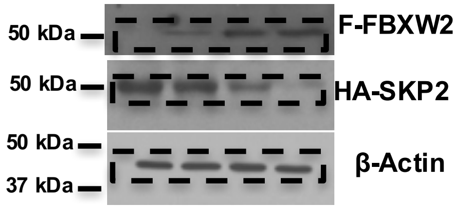

Figure s4e

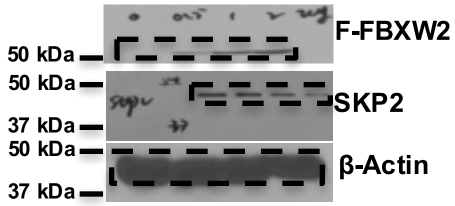

Figure s4f

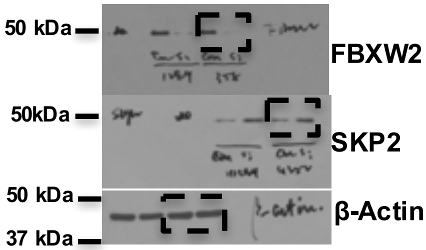

Figure s4g

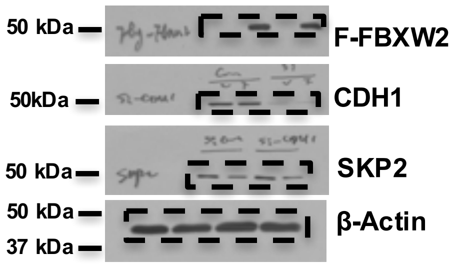

Figure s4h

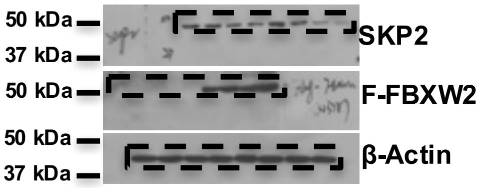

Figure s4i

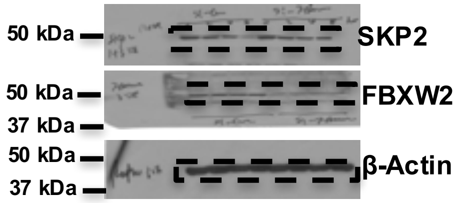

Figure s4j

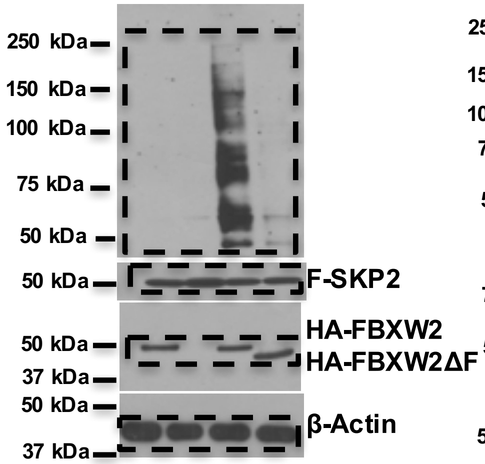

Figure s4k

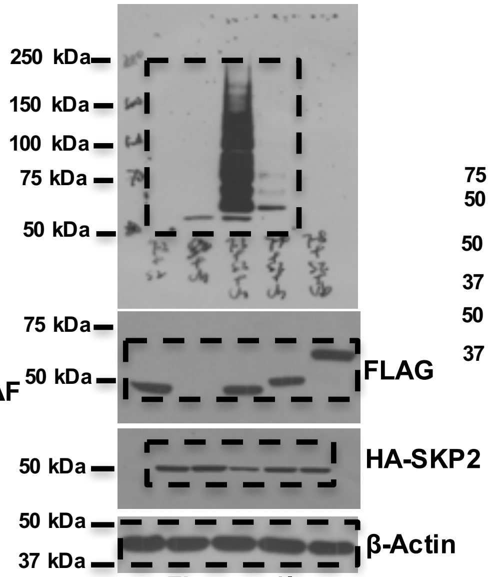

Figure s4l

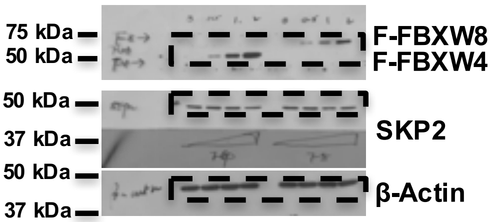

Figure s4m

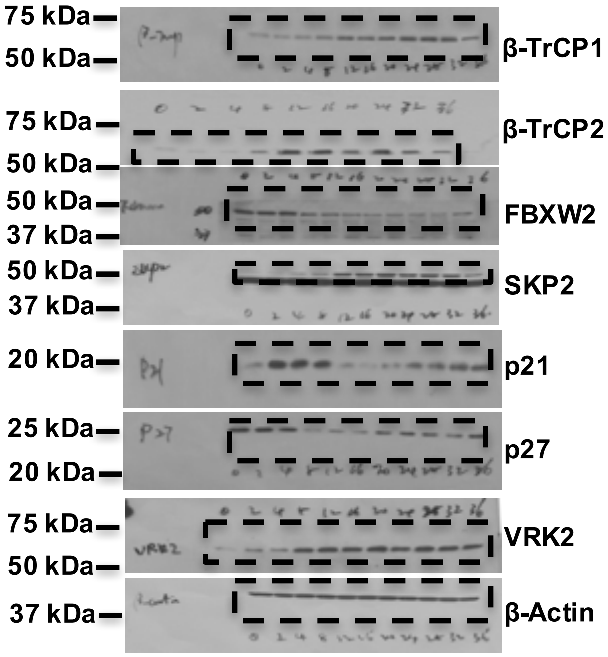

Figure 5a

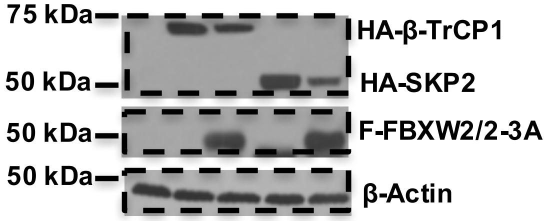

Figure 5b

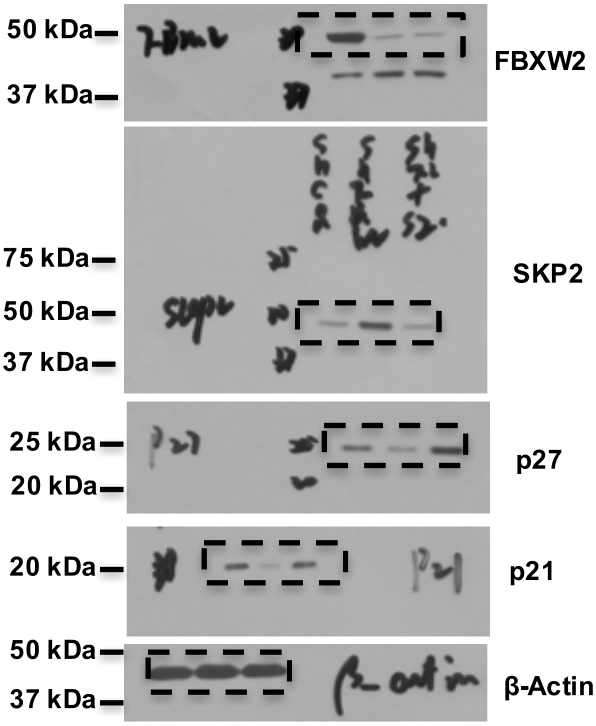

Figure 5g

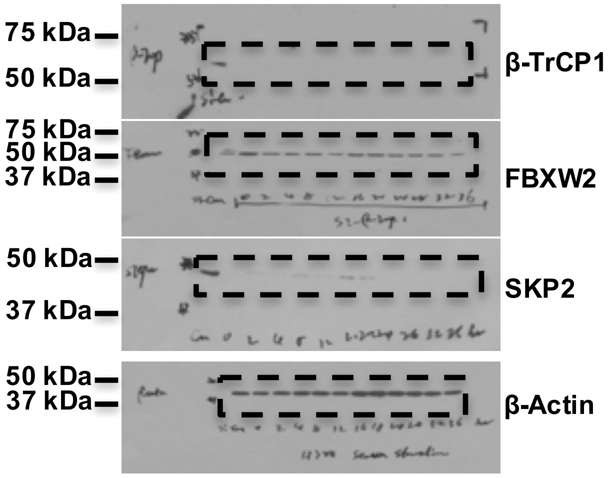

Figure s5a

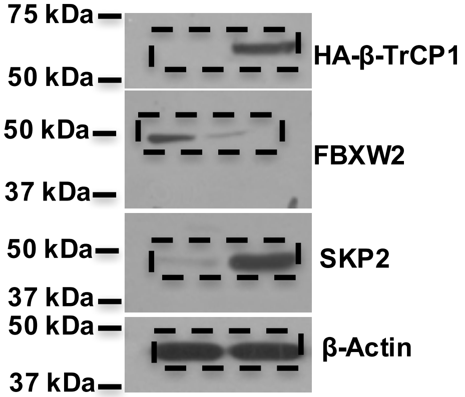

Figure s5b

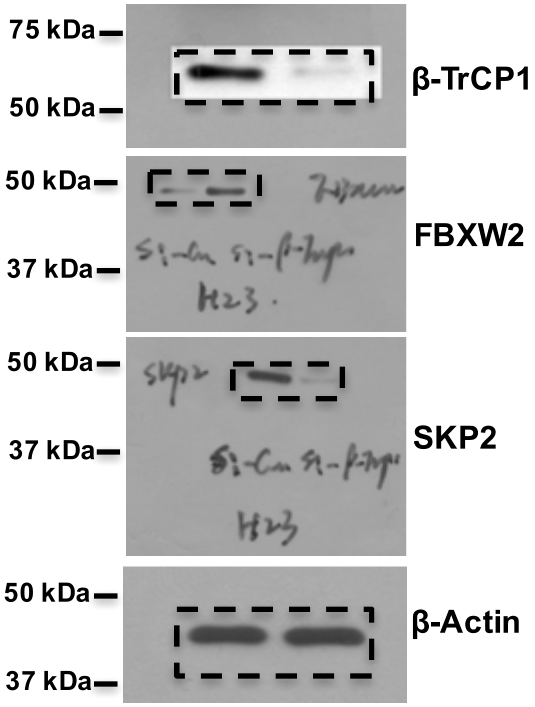

Figure s5c

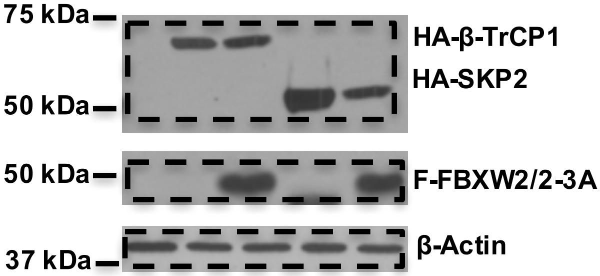

Figure s5d

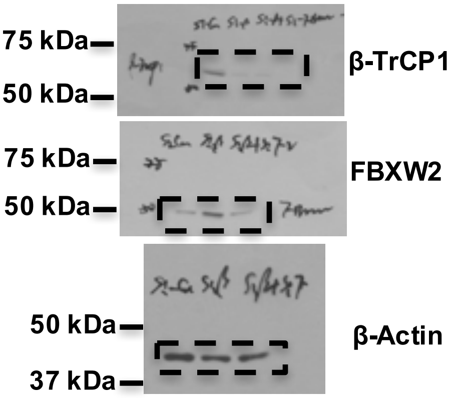

Figure s5i

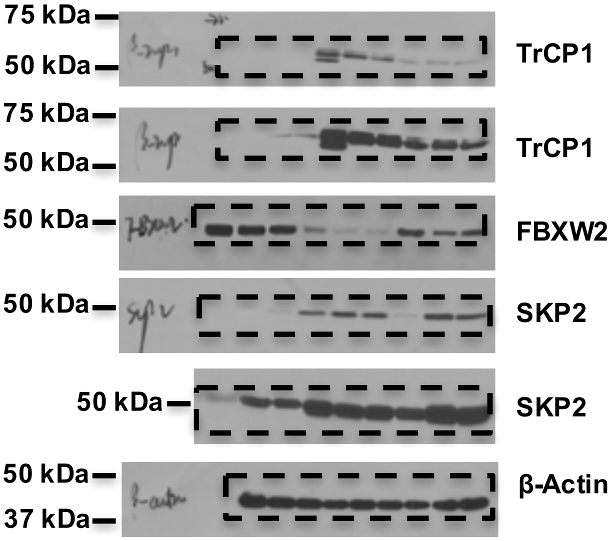

Figure 6a

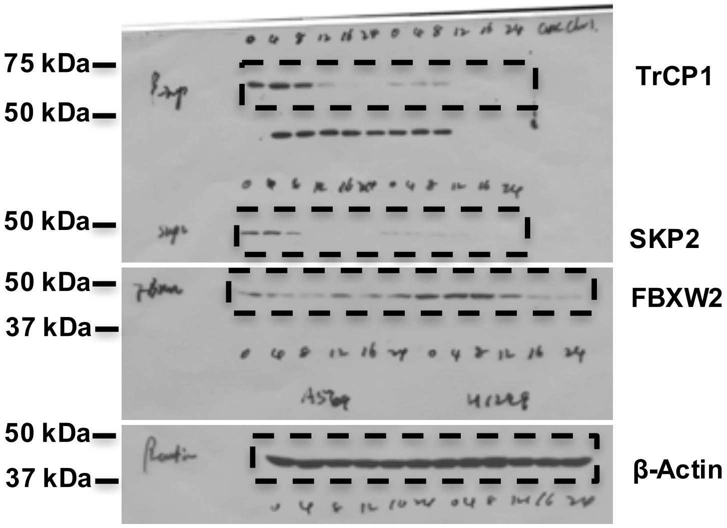

Figure s6b

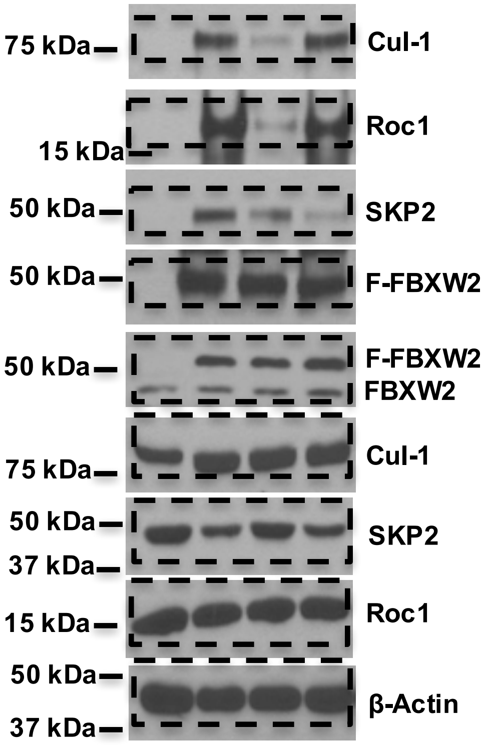

Figure 7a

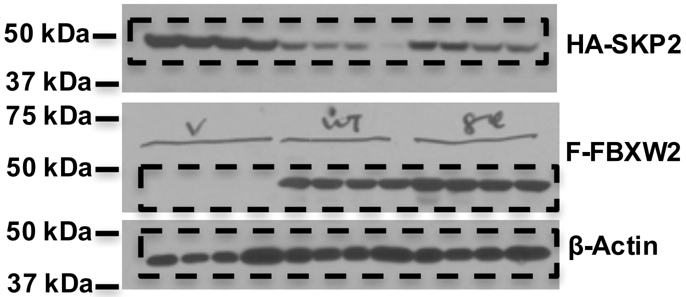

Figure 7b

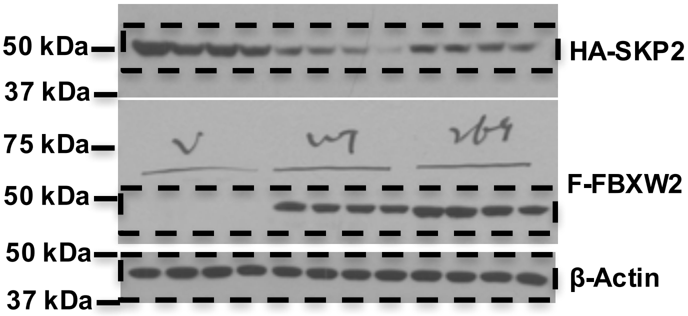

Figure 7c

Supplementary Figure 8. Original X-ray films for immunoblotting analysis: Shown are all original X-ray films for immunoblotting analysis included in this study. The blot areas within the dotted boxes are shown in the figures and supplementary figures, as indicated.

Supplementary Table 1. Putative kinases for FBXW2 phosphorylation at the β-TrCP binding motif (SSGART).

Position	Code	Kinase	Peptide	Score	Cutoff
6	S	TKL	**ACWEWSSGARTQH	8.028	4.148
7	S	AGC/GRK	*ACWEWSSGARTQHF	14.767	7.991
11	T	AGC/GRK	EWSSGARTQHFRG**	8.026	7.991
11	T	AGC/PKC	EWSSGARTQHFRG**	1.989	1.416
7	S	CK1/CK1	*ACWEWSSGARTQHF	7.485	3.998
6	S	CK1/VRK	**ACWEWSSGARTQH	5.2	0.997
7	S	CK1/VRK	*ACWEWSSGARTQHF	3.9	0.997
11	T	CK1/VRK	EWSSGARTQHFRG**	3.2	0.997
11	T	Other/Haspin	EWSSGARTQHFRG**	11	9
11	T	Other/PEK	EWSSGARTQHFRG**	5.636	4.722
11	T	Other/TLK	EWSSGARTQHFRG**	2.75	2.312
6	S	TKL/IRAK	**ACWEWSSGARTQH	2.5	2.088
11	T	TKL/IRAK	EWSSGARTQHFRG**	2.5	2.088
7	S	TKL/MLK	*ACWEWSSGARTQHF	11.08	9.761
6	S	AGC/GRK/BARK	**ACWEWSSGARTQH	7.023	4.78
7	S	AGC/GRK/BARK	*ACWEWSSGARTQHF	10.674	4.78
11	T	AGC/GRK/BARK	EWSSGARTQHFRG**	10.884	4.78
7	S	AGC/PKC/PKCa	*ACWEWSSGARTQHF	7.126	4.803
7	S	CK1/CK1/CK1-A	*ACWEWSSGARTQHF	4.579	3.887
6	S	CK1/VRK/VRK2	**ACWEWSSGARTQH	18.25	5.225
7	S	CK1/VRK/VRK2	*ACWEWSSGARTQHF	18.5	5.225
11	T	CK1/VRK/VRK2	EWSSGARTQHFRG**	15.5	5.225
7	S	CMGC/CDK/CDK9	*ACWEWSSGARTQHF	5.2	4.647
11	T	Other/Haspin/Haspin	EWSSGARTQHFRG**	11	9

7	S	STE/STE20/MSN	*ACWEWSSGARTQHF	5.5	3.05
11	T	TKL/IRAK/IRAK1	EWSSGARTQHFRG**	3.75	2.8
7	S	TKL/LISK/LIMK	*ACWEWSSGARTQHF	11	9
6	S	AGC/GRK/BARK/BARK1	**ACWEWSSGARTQH	15.31	5.269
7	S	AGC/GRK/BARK/BARK1	*ACWEWSSGARTQHF	10.095	5.269
11	T	AGC/GRK/BARK/BARK1	EWSSGARTQHFRG**	23.286	5.269
6	S	AGC/PKC/PKCa/PRKCA	**ACWEWSSGARTQH	9.187	7.372
7	S	AGC/PKC/PKCa/PRKCA	*ACWEWSSGARTQHF	16.091	7.372
11	T	AGC/PKC/PKCa/PRKCA	EWSSGARTQHFRG**	10.525	7.372
11	T	AGC/PKC/PKCi/PRKCI	EWSSGARTQHFRG**	7	6.256
7	S	Atypical/PDHK/PDHK/PDK1	*ACWEWSSGARTQHF	17.108	4.74
7	S	CAMK/CAMKL/AMPK/PRKAB1	*ACWEWSSGARTQHF	3.333	2.525
7	S	CAMK/CAMKL/QIK/SIK1	*ACWEWSSGARTQHF	5	4.074
11	T	CMGC/CDK/CDK7/CDK7	EWSSGARTQHFRG**	0.939	0.406
6	S	CMGC/MAPK/ERK/Erk3	**ACWEWSSGARTQH	2.25	2.088
7	S	Other/ULK/ULK/ULK3	*ACWEWSSGARTQHF	4	3.15
7	S	STE/STE20/FRAY/OSR1	*ACWEWSSGARTQHF	1.429	1.307
11	T	STE/STE20/FRAY/OSR1	EWSSGARTQHFRG**	2.857	1.307
6	S	STE/STE7/MEK3/MAP2K3	**ACWEWSSGARTQH	4.889	1.307
6	S	STE/STE7/MEK3/MAP2K4	**ACWEWSSGARTQH	5.2	1.325
7	S	TKL/LISK/LIMK/LIMK1	*ACWEWSSGARTQHF	11	9

Position: The position of the site that is predicted to be phosphorylated. **Code**: The residue that is predicted to be phosphorylated. **Kinase**: The regulatory kinase that is predicted to phosphorylate the site. **Peptide**: The predicted phosphopeptide with 7 amino acids upstream and 7 amino acids downstream around the modified residue. **Score**: The value calculated by GPS algorithm (http://gps.biocuckoo.org) to evaluate the potential of phosphorylation. The higher the value, the more potential the residue is phosphorylated. **Cutoff**: The cutoff value under the threshold. Different threshold means different precision, sensitivity and specificity.
